# Distinct Survival Outcomes in Subgroups of Stage III Pancreatic Cancer Patients: Taiwan Cancer Registry and Surveillance, Epidemiology and End Results registry

**DOI:** 10.1245/s10434-021-11030-w

**Published:** 2021-11-13

**Authors:** Tzu-Pin Lu, Chien-Hui Wu, Chia-Chen Chang, Han-Ching Chan, Amrita Chattopadhyay, Wen-Chung Lee, Chun-Ju Chiang, Hsin-Ying Lee, Yu-Wen Tien

**Affiliations:** 1 Department of Public Health, College of Public Health, Institute of Epidemiology and Preventive Medicine, National Taiwan University, Taipei, Taiwan; 2 Department of Surgery, National Taiwan University Hospital, Taipei, Taiwan; 3 Taiwan Cancer Registry, Taipei, Taiwan

## Abstract

**Purpose:**

Pancreatic cancer is one of the most malignant cancers with poor survival. The latest edition of the American Joint Committee on Cancer (AJCC) staging system classifies the majority of operable pancreatic cancer patients as stage-III, while dramatic heterogeneity is observed among these patients. Therefore, subgrouping is required to accurately predict their prognosis and define a treatment plan. This study conducts a cohort study to provide a more precise classification system for stage-III pancreatic cancer patients by utilizing clinical variables.

**Methods:**

We analyzed survival using log-rank tests, univariate Cox-regression models, and Kaplan-Meier survival curves for stage-III pancreatic ductal adenocarcinoma (PDAC) patients from the Taiwan Cancer Registry (TCR). Patients were further divided into subgroups using classification and regression tree (CART) algorithm. All results were validated using the SEER database.

**Results:**

Among stage-III PDAC patients, lymph node and tumor grade showed significant association with survival. Patients with N2 stage had higher mortality risks (hazard ratio [HR] = 2.30, 95% confidence interval [CI] 1.71–3.08, *p* < 0.0001) than N0 patients. Patients with grade 3 also had higher risk of mortality (HR = 3.80, 95% CI 2.25–6.39, *p *< 0.0001) than grade 1 patients. The CART algorithm stratified stage-III patients into four subgroups with significantly different survival rates. The median survival of the four subgroups was 23.5, 18.4, 14.5, and 9.0 months, respectively (*p *< 0.0001). Similar results were observed with SEER data.

**Conclusions:**

Lymph node involvement and tumor grade are predictive factors for survival in stage-III PDAC patients. This new precise classification system can be used to guide treatment planning in advanced-stage pancreatic cancer.

**Supplementary Information:**

The online version contains supplementary material available at 10.1245/s10434-021-11030-w.

Pancreatic cancer has a dismal prognosis, and its overall 5-year survival rate is less than 10%.^1,2^ Only 20% of pancreatic cancer patients are diagnosed at a stage where they can receive potentially curative surgery while the other 80% of patients must rely on chemotherapy only.^3^ In 2020, 57,600 pancreatic cancer cases were diagnosed, and 47,050 deaths occurred in the United States.^4^ In Taiwan, an Asian country with a population of 2.3 million, approximately 2000 newly diagnosed cases of pancreatic cancer are reported each year. The proportion of pancreatic cancer patients who are eligible for surgery is 20–25% in Taiwan, which is comparable to the statistics for the rest of the world.^5^

Over the past few decades, the American Joint Committee on Cancer (AJCC) has established a system for cancer staging based on three key components: local tumor extent (T stage); dissemination to the regional lymph nodes (N stage); and metastatic spread to distant sites (M stage), known as TNM staging.^6^ The TNM staging system for pancreatic cancer has some limitations, which are difficult to overcome because of the nature of the disease. First, pancreatic cancer has many subtypes, which include pancreatic ductal adenocarcinoma (PDAC), pancreatic neuroendocrine tumor/carcinoma, intraductal papillary mucinous carcinoma, and others. Approximately 90% of all pancreatic cancer patients have the PDAC subtype, whereas the remaining 10% are diagnosed with other subtypes. The most important difference among the subtypes is in their survival rate. Second, the resection margin status of pancreatic cancer may affect the survival, which is not considered within the TNM staging system. Third, the TNM staging system is largely based on single-institution studies in high-volume academic centers catering to a homogeneous patient population, which limits its generalizability to other settings. Fourth, it is difficult to classify the neoadjuvant cohort using the staging system. Almost all recent studies based on TNM staging excluded patients who received neoadjuvant therapy. Although these disadvantages limit TNM’s clinical applicability in daily practice, nevertheless, it is widely used for predicting prognosis and exchanging cancer information.

The 8th edition of the AJCC staging manual (AJCC 8), published in 2016, is widely used in both the United States (since 2018) and Taiwan (since 2019). In this revised staging system, the T-category was classified by size, regardless of the extrapancreatic invasion. The N-category was modified from N0/N1 to N0/N1/N2 leading to a more equal distribution of patients among stages and an increased prognostic accuracy.^7–11^ However, when closely evaluated, more operable patients with PDAC (15–20% of the total pancreatic cancer patients) are classified into stage III in AJCC 8,^11,12^ in part because the 8th edition defines stage III as either T4 or N2, both of which are common in PDAC. Therefore, prediction precision of survival outcomes is compromised when such a substantial proportion of operable patients is classified as stage III. This makes diagnosis of stage III more controversial,^13^ in terms of survival and treatment due to (a) lack of consistency of the definition of resectability (borderline resectable or locally advanced), and (b) differing treatment strategies, including the surgical techniques for the locally advanced disease, across institutions. For such an advanced disease state, physicians tend to ignore the tumor grade, which represents the aggressive biology of the tumor itself and has been widely confirmed as a prognostic factor in pancreatic cancer.^14^ To address these issues, a subgrouping of stage III PDAC patients is required to accurately predict their prognosis and then to define the treatment plan.^15^

In this study, we aimed to provide a more precise classification system by utilizing clinical variables. We retrieved the clinical variables and survival outcomes of the pancreatic cancer patients in the Taiwan Cancer Registry (TCR). Univariate Cox proportional hazard regression modeling and a classification and regression tree (CART) algorithm were used to identify variables significantly predictive of survival. The four subgroups stratified by tumor grade and N stage showed significantly different survival outcomes. The same subgrouping strategy showed consistent and stable results in the Surveillance, Epidemiology, and End Results (SEER) database.

## Methods

### Study Subjects in Taiwan as the Identification Set

The TCR is a national registration system established in 1979 by the Ministry of Health and Welfare in Taiwan to deposit core information for cancer patients.^5,12,16^ It is constructed with data of all cancer sites from newly diagnosed malignant cancer patients over the past 40 years. In general, the TCR, with a 98.4% coverage, 93% histological verification, and 97.6% morphological verification (excluding liver), is the most important cancer registry in Taiwan.^17,18^ Pancreatic cancer has an estimated coverage rate of approximately 60% for detailed cancer staging parameters in Taiwan since 2013. Admittedly, however, some limitations exist in the TCR, because some clinical factors and surgical variables may be missing or not documented in the database. Pancreatic cancer patients diagnosed from January 1, 2013 to December 31, 2017 were retrieved from TCR and included as the primary study subjects (N = 5,685) in this study. The original staging information recorded in the TCR and the SEER databases used AJCC7, and we utilized the following procedures to transform the staging according to AJCC8. The TNM definitions for PDAC patients in both AJCC7 and AJCC8 are shown in Supplementary Table S1. For the metastasis (M) stage, its definition is the same in both versions, thus no changes were made. For the tumor size (T) stage, the patients labeled with T2 in AJCC7 were reclassified into T2 or T3 for AJCC8 based on the tumor size (≤40 mm or >40 mm). The patients labeled with T3 and T4 in AJCC7 were integrated into T4 for AJCC8. For the regional lymph node (N) stage, the patients labeled with N1 in AJCC7 were reclassified into N1 or N2 for AJCC8 based on the number of lymph nodes involved (1-3 nodes or ≥4 nodes). Lastly, we determined the stage of cancer based on these transformed TNM stages (Supplementary Table S2).

**Fig. 1:**
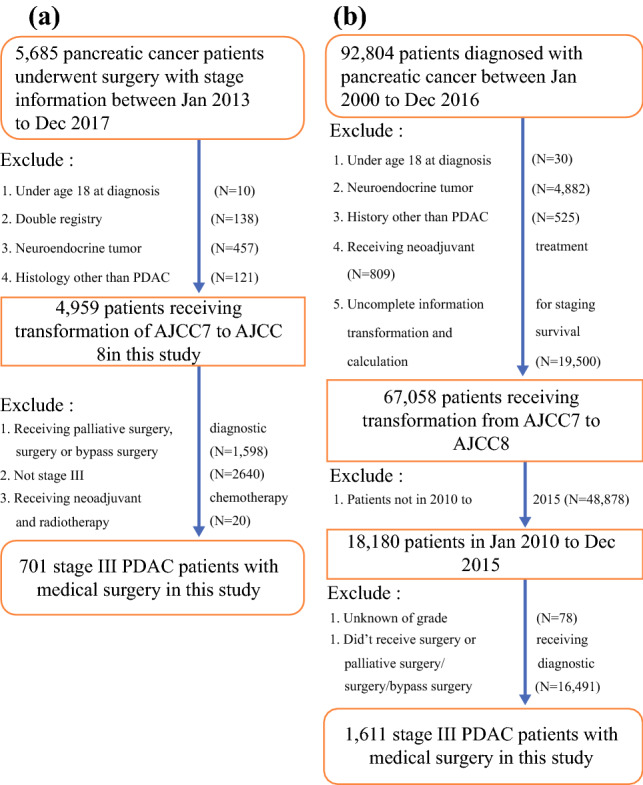
Patient selection and exclusion criteria utilized for datasets: **a** Taiwan Cancer Registry database, and **b** SEER database

The flowchart in Fig. 1a illustrates the inclusion–exclusion criteria in this study. First, only patients with PDAC were retained, resulting in 578 patients being excluded due to different histological types. These were 457 patients with pancreatic neuroendocrine tumor (C25.4) and 121 patients with histological types other than PDAC. Only ICD codes C25.0–3 and C25.7–9 were included in this study. Second, we excluded patients with palliative bypass surgery or diagnostic surgery (*N *= 1598) and patients who were not stage 3 disease (*N *= 2640). Third, patients receiving other treatments before the surgery, such as neoadjuvant radiation therapy and neoadjuvant chemotherapy, were removed to reduce the heterogeneity (*N *= 20). Lastly, only 701 patients with definite surgery information and clear clinical variables, including tumor size and stage information, were analyzed (Fig. 1a). This study has been approved by the institutional review boards of National Taiwan University Hospital (201910027W).

### Study Subjects from the SEER Data as the Validation Set

The SEER database was used as the replication set in this study.^19^ A total of 92,804 pancreatic cancer patients were recorded in the SEER dataset (Fig. 1b). To match the study time period with that of TCR, we only analyzed the pancreatic cancer patients with PDAC subtype (patients with pNET and histological types other than PDAC were excluded) diagnosed from January 1, 2010 to December 31, 2015 in the SEER dataset. Applying same inclusion–exclusion criteria, as described for TCR (Fig. 1b), we kept PDAC patients with definite surgery status and clinical variables for further analysis (*N *= 1611).

### Statistical Analysis

Two statistical methods, including the log-rank test and Cox proportional hazards regression modeling, were utilized to evaluate the association of clinical variables and survival in PDAC patients, using an exponential distribution (“rpart” in R, using “method=exp”). The survival data are illustrated using Kaplan-Meier survival curves. The CART algorithm, a binary tree established by a recursive method, was performed (rpart package in R) to determine the best variable and the corresponding cutoff value to dichotomize the samples into two groups with the largest differences in survival.^20^ The CART algorithm repeatedly estimates the Gini index of each variable and the cutoff value in order to identify the subgrouping that maximizes the difference in the survival outcomes using a Poisson model.^21^ An evaluation of ten-fold cross-validation was conducted to ensure no overfitting using TCR for ten trials. Finally, the concordance probability (C-Index) and the time-dependent AUC (area under ROC curves) were measured to judge prognostic importance of the model for both TCR and SEER.^22,23^

## Results

### Patient Characteristics: Taiwan Cancer Registry Database

A total of 701 PDAC patients from the TCR database met the inclusion criteria and were included in this study (Fig. 1). The staging information for these patients was transformed from AJCC7 to AJCC8 using the procedures described above. In general, the survival outcomes of the PDAC patients followed the severity of the staging information in AJCC8. However, more patients were classified as stage III in AJCC8 than AJCC7, which results in difficulty in precisely classifying the patients based on their mortality risk.

### Survival Analysis using Taiwan Cancer Registry Database

To evaluate whether the stage III PDAC patients can be further divided into subgroups, we used a univariate Cox proportional hazard regression model to assess the clinical variables (Table 1a). The detailed summary characteristics of the significant variables of the participants from TCR is provided in Supplementary Table S3. As shown in Table 1, tumor grade, N stage, and chemotherapy were all significant predictors of survival (*p *< 0.05). Higher tumor grade and higher number of N stage led to significantly worse survival outcomes, while receipt of chemotherapy significantly improved the odds of survival. Because chemotherapy is a treatment option instead of an intrinsic tumor characteristic, we focused on tumor grade and N stage to further classify the stage III PDAC patients.

**Table 1:** Results of univariate Cox hazard regression model in the stage III PDAC patients from the TCR database

Variable	Coding	HR	95% CI	*p* value
Age		1.01	0.99-1.01	0.08
Tumor size		1.00	0.99-1.00	0.44
Sex	Male	–	–	–
	Female	0.99	0.80–1.22	0.96
Grade	0	–	–	–
	1	1.70	1.03–2.79	0.04*
	2	3.80	2.25–6.39	<0.0001*
Lymph node involvement (N)	0	–	–	–
	1	1.52	1.18–1.94	<0.0001*
	2	2.30	1.71–3.08	<0.0001*
Chemotherapy	No	–	–	––
	Yes	0.58	0.46–0.72	<0.0001*
Radiotherapy	No	–	–	–
	Yes	0.99	0.70–1.36	0.93

*HR* hazard ratio; *CI* confidence interval

*Significant (*p *< 0.05)

Dependent variable in the regression model was time to death

To evaluate whether tumor grade and N stage have synergistic effects on survival, we classified the patients into nine subgroups (Supplementary Table S4) and plotted their survival in Kaplan-Meier curves (Supplementary Fig. S1). In general, patients with lower grade and fewer lymph nodes involved, such as N0-Grade1 and N1-Grade1, showed better survival outcomes, whereas higher grade and more lymph node involvement, such as N1-Grade3 and N2-Grade3 led to poor survival outcomes. We further examined whether the survival outcomes exhibited any significant differences by using a log-rank test among the nine subgroups (Supplementary Table S5). In patients with a medium tumor grade (Grade 2), survival showed significant differences depending on their N stage, whereas in patients with low (Grade 1) or high tumor grade (Grade 3), the degree of lymph node involvement did not significantly affect their survival (Supplementary Table S5). Together, these results suggest that tumor grade information should be taken into consideration when predicting the prognosis in stage III PDAC patients.

**Fig. 2:**
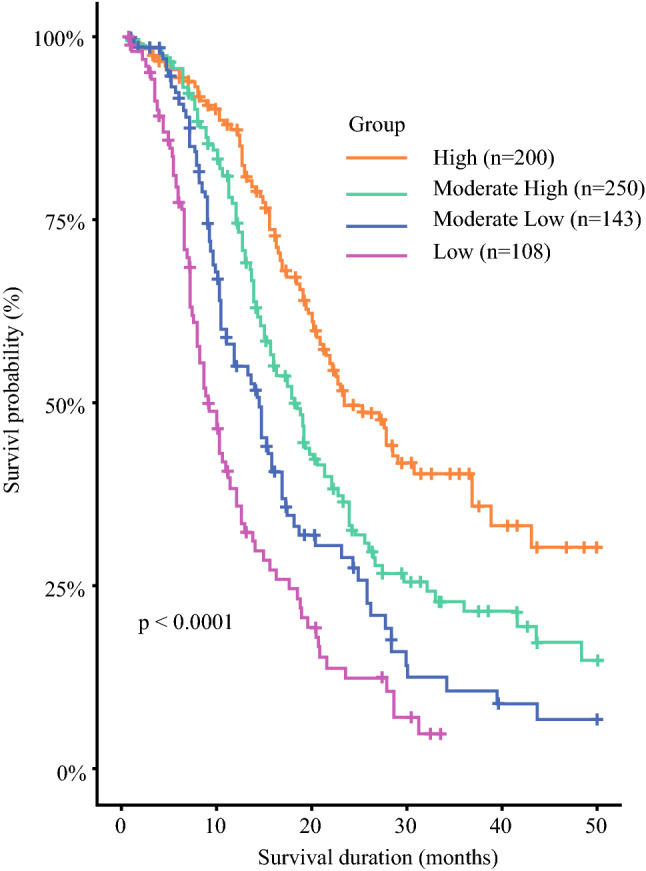
Survival curves of the four subgroups generated from tumor grade and N stage in the TCR database. The x-axis denotes the survival duration in months, and the y-axis denotes the survival probability in percentage. The *p*-value was obtained from the log-rank test

For real-world applications, nine is a large number of subgroups; thus, we used the CART algorithm to combine the subgroups with similar survival outcomes. First, we performed the CART algorithm to analyze all available clinical variables shown in Table 1. Unsurprisingly, the two most important variables for subgroup were tumor grade and N stage, which concurred with the results of our previous univariate analysis. Next, we utilized the CART algorithm with the nine subgroups derived from tumor grade and N stage, which generated four groups corresponding to predictions of high, moderately high, moderately low, and low survival (Supplementary Fig. S2). To ensure that there was no overfitting in the final classified tree, ten trials of tenfold cross-validation analyses were conducted using CART for the TCR data. The chosen classification tree with four major subgroups (high, moderately high, moderately low, and low survival) was observed in approximately 50–60% of the subsets, suggesting its reproducibility and stability. The Kaplan-Meier survival curves for these four groups are illustrated in Fig. 2. Notably, these patients showed significant differences in the survival outcomes after being reclassified into four subgroups (*P* < 0.0001). Across the four subgroups, the median survival duration was 23.5 months in the subgroup with the highest survival rates, whereas the median survival duration was only 9 months in the subgroup with the lowest survival rates (Table 2). In addition, the Cox proportional hazards regression analysis demonstrated patients with distinct survival probabilities in the four subgroups (Table 2). Furthermore, another tenfold cross-validation analysis was conducted using the TCR data to gauge the efficacy of the model. The average c-index of the model over tenfold cross-validations was reported to be 0.68 with a standard deviation of 0.007 and the average time-dependent AUC from 1 year to 4 years were 0.72, 0.65, 0.69, and 0.65, respectively (Supplementary Figure S3) with standard deviations of 0.009, 0.011, 0.017 and 0.020, respectively. Both the c-index and time-dependent AUC were comparable to that from the published reports on PDAC using large cancer registries.^24,25^ These results suggest that it is necessary to consider the heterogeneous nature of stage III PDAC patients rather than treating them as a homogeneous single group.

**Table 2:** Statistics of the median survival duration and Cox hazard regression model of the four subgroups stratified by tumor grade and N stage in the TCR database

Subgroup	N	Median survival duration (95% CI)	HR (95% CI)	*p* value*
High	200	23.5 (21–∞)	Ref	Ref
Moderately high	250	18.4 (15.8–21.2)	1.63 (1.23–2.15)	0.0009
Moderately low	143	14.5 (11.2–16.9)	2.46 (1.81–3.37)	< 0.0001
Low	108	9.0 (7.9–11.4)	4.13 (3.01–5.68)	< 0.0001

*HR* hazard ratio; *CI* confidence interval

*p value was obtained from the Cox hazard regression model

Dependent variable in the regression model was time to death

### Replication Analysis Using SEER Database

**Fig. 3:**
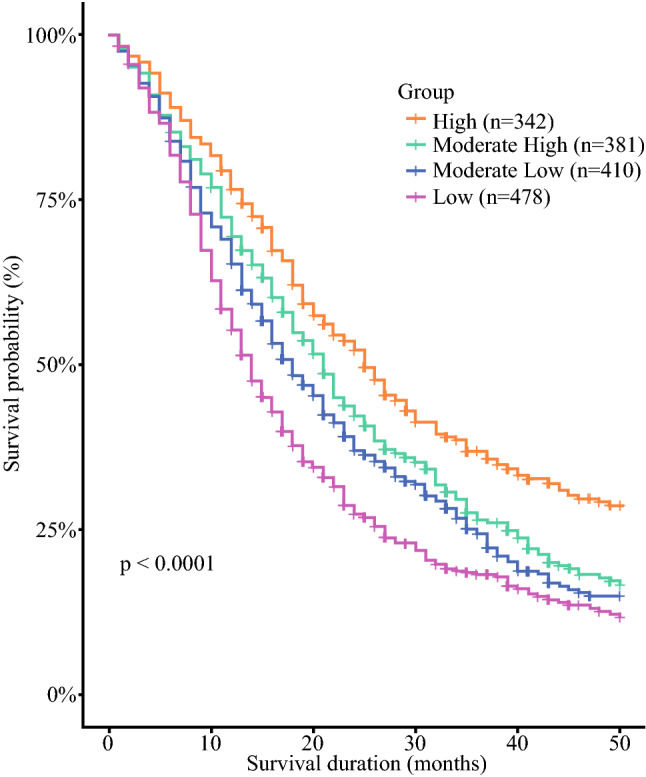
Survival curves of the four subgroups generated from tumor grade and N stage in the SEER database. The x-axis denotes the survival duration in months, and the y-axis denotes the survival probability in percentage. *P*-value was obtained from the log-rank test

To evaluate whether these findings are reproducible in other datasets, we retrieved data on PDAC patients from the SEER database. Using the exclusion criteria described in Methods, a total of 1611 stage III PDAC patients were analyzed in this part of the study (Fig. 1b). First, we examined the association of each clinical variable with the survival outcome for the samples from the SEER database. Similar to the results obtained from the TCR database, tumor grade and N stage were found to be significant predictors of survival in the stage III PDAC patients in the SEER database (*p *< 0.0001). The C-index (0.67) and the time-dependent AUC from 1 year to 4 years (0.60, 0.61, 0.59, and 0.62) also concurred with that of TCR and other prior published studies on PDAC from large cancer registries.^24,25^ Notably, the Kaplan-Meier survival curves of the nine subgroups obtained from the two variables, tumor grade and N stage, showed clear survival trends in the SEER data (Supplementary Figure S4), suggesting that these two variables remain important predictors across distinct ethnic backgrounds. Subsequently, we reclassified the nine subgroups into the four subgroups determined by the CART algorithm (Supplementary Fig. S2). As shown in Fig. 3, the pattern of results from the SEER database were replicable to those from the TCR database (Fig. 2). The survival for each of the four subgroups were further, comparatively plotted, as shown in Supplementary Fig. S5. Survival outcomes between TCR and SEER were observed to have no significant difference for the high and moderately high survival subgroups, whereas significant differences were observed in the moderately low and low survival subgroups (*p *< 0.05). The results of the Cox proportional hazards regression model in the four subgroups from the SEER database also concurred with the results obtained from the TCR database (Supplementary Table S6). In conclusion, tumor grade and N stage can be used to further stratify stage III PDAC patients into four subgroups corresponding to their predicted survival. This finding applied to both the TCR database with patients of Asian ancestry and the SEER database with patients of European ancestry.

## Discussion

PDAC is a highly lethal malignant tumor type, and surgery has been shown as the most effective treatment to prolong survival in PDAC patients.^9^ However, a previous study showed that while surgery is important, it is not enough to treat PDAC patients.^26^ One possible solution to manage PDAC patients is to add neoadjuvant treatments to increase the number of patients eligible to receive surgery.^21^ Another approach is to use adjuvant chemotherapy to treat PDAC patient after surgery, and such an approach has been shown to be an effective treatment in several previous studies and this study (Table 1).^27–29^ However, it is prerequisite to provide an accurate estimation of the prognosis of PDAC prior to designing the treatment plans of chemotherapy. The most popular cancer staging system for PDAC is AJCC8, and it has been widely validated in international studies.^6,9,11^ However, the uneven distribution of the samples in different stages of PDAC in AJCC8 poses a major challenge in designing the treatment plans for stage III PDAC patients. To the best of our knowledge, this issue has not been carefully addressed before.

Stage III pancreatic cancer is composed of many T4 tumors, for which neoadjuvant remains as the primary treatment approach.^30^ However, because people respond differently to neoadjuvant treatment, T4 tumor patients who received neoadjuvant therapy may have completely different posttreatment and survival results. It is important to discuss and analyze locally advanced stage III cases; however, neoadjuvant treatment was not a popular first-choice, in the inclusion period for TCR. Therefore, due to lack of data, it was not possible to analyze this population in this study. Preoperative downstaging chemotherapy for locally advanced pancreatic cancer (LAPC) began in 2018 in Taiwan, and the screening period was set from 2013 to 2017 to exclude patients with neoadjuvant chemotherapy. Therefore, there were no data for LAPC patients undergoing surgery after downstaging chemotherapy. Downstage by preoperative chemotherapy will increase margin negative resection and survival in patients with LAPC. Selection of patients, with good response to chemotherapy and without metastasis during the chemotherapy period, for surgery, is one of the major factors for higher margin-negative resection and better patients’ survival. Vascular involvement in LAPC is not uniform and different involvement patterns have major implications for the surgical management and quality of resection. A lot of studies focusing on downstaging postneoadjuvant therapy exists in the literature.^31–34^ If patients responded well to neoadjuvant therapy, resectability increased, and if they had the opportunity to undergo surgery, such patients could even survive beyond the usual survival time of the first- or second-stage patients. If their response to neoadjuvant therapy was only mediocre, resectability remained unchanged. Even if there was no way to receive surgery, local treatment, including radiotherapy, might have better survival rate than that of some N2-stage III patients. However, patients with poor responses to chemotherapy might have the same survival rate as that of stage IV patients. As for the effect of neoadjuvant treatment on the subgroups of stage III PDAC, no large-scale study exists that provides evidence of neoadjuvant therapy affecting change of tumor grade. The N status is very likely to downgrade because of neoadjuvant therapy, but the T status may not necessarily change. Therefore, there remains a possibility that using the classification method to identify patients with N0 (compared with N1 N2) and G1 (compared with G2 G3), with better prognosis, and administer neoadjuvant treatment with surgery would lead to better survival rate.

A univariate Cox proportional hazards regression model indicated that tumor grade and N stage were significant predictors of survival in stage III PDAC patients (Table 1), which also was validated using the CART algorithm. Using tumor grade and N stage, an initial set of nine subgroups and then a set of four subgroups were identified in both the TCR and the SEER database (Supplementary Figs. S1–S2, S4). It is not surprising that N stage is a significant predictor of survival, because N stage has already been utilized in the AJCC8. However, tumor grade is not taken into consideration in the AJCC8. Our results demonstrated that a stage III PDAC patient showed poor survival outcomes when the tumor grade is advanced, and thus it is necessary to incorporate tumor grade into the prediction system of stage III PDAC patients in the future.

Some limitations exist in this study. First, the sample size of the stage III PDAC patients is relatively small in the TCR database. However, the classification strategy using tumor grade and N stage were consistent with and replicated in the SEER database, which has a large sample size. Therefore, the results suggest that the classification scheme established in this study to predict prognosis of stage III PDAC patients is stable. Second, the TCR is a nationwide cancer registry, and its data were collected from different hospitals in Taiwan. Consequently, missing values exist in those clinical variables, and the data quality must be assessed and evaluated before performing a comprehensive analysis. Previous studies have demonstrated that the TCR is a useful cancer registry with high quality of deposited data.^5,12^ We have developed an online prediction system for breast cancer patients in Taiwan by using the TCR database.^16^ Finally, the longest follow-up time of the PDAC patients in the TCR database was less than 5 years in this study. However, considering the rapid progression and high mortality rate of PDAC, the follow-up time from the TCR database was sufficient to perform an analysis of the survival outcomes. Notably, the survival probabilities of the stage III PDAC patients were comparable in both the TCR and the SEER database (Figs. 2, 3).

## Conclusions

Our findings demonstrate that stage III PDAC patients can be divided into four subgroups with distinct survival outcomes and that the results of our classification approach were consistent across two large-scale cancer registries with different genetic backgrounds. These results may be used to inform treatment planning in the future for patients diagnosed with stage III PDAC.

## Supplementary Information

Below is the link to the electronic supplementary material.

Supplementary file1 (DOCX 901 kb)
