# Supplementary material for: Distinct Survival Outcomes in Subgroups of Stage III Pancreatic Cancer Patients: Taiwan Cancer Registry and Surveillance, Epidemiology and End Results registry
Source: Ann Surg Oncol. 2021 Nov 13;29(3):1608–15. doi: 10.1245/s10434-021-11030-w (PMC8810458; doi:10.1245/s10434-021-11030-w)
Supplement: Supplementary file 1 — Supplementary file1 (DOCX 901 kb) [file 10434_2021_11030_MOESM1_ESM.docx]

**Supplementary Data File**

**Distinct survival outcomes in subgroups of stage III pancreatic cancer patients: Taiwan Cancer Registry and Surveillance, Epidemiology and End Results registry**

Tzu-Pin Lu^1,2^, Chien-Hui Wu^1,2^, Chia-Chen Chang.^1,#^, Han-Ching Chan^1,#^, Amrita Chattopadhyay^1^, Wen-Chung Lee^1,3^, Chun-Ju Chiang^1,3^, Hsin-Ying Lee^1^, Yu-Wen Tien^2*^

^1^ Institute of Epidemiology and Preventive Medicine, Department of Public Health, College of Public Health, National Taiwan University, Taipei, Taiwan

^2^ Department of Surgery, National Taiwan University Hospital, Taipei, Taiwan

^3^ Taiwan Cancer Registry, Taipei, Taiwan

^#^ Equal-contributions

**Running Title:** Survival outcomes: Stage III PDAC

**Conflict of Interest Statement:** The authors declare no potential conflicts of interest.

**Declarations of commercial interest:** none

**Financial Support:** This study was supported by grants from the Health Promotion Administration, Ministry of Health and Welfare, Taipei, Taiwan, grant no. A1081115: Tobacco Health and Welfare Taxation, and the Ministry of Science and Technology, Taiwan (MOST-106-2314-B-002-134-MY2, MOST-108-2314-B-002-103-MY2, and MOST-109-2314-B-002-151-MY3). The content of this research may not represent the opinion of the Health Promotion Administration, Ministry of Health and Welfare. The funders had no role in the study design, data collection and analysis, the decision to publish, or preparation of the manuscript.

*** Corresponding author:**

Yu-Wen Tien, MD, PhD

Department of Surgery

National Taiwan University Hospital

No. 7 Chung-Shan South Rd

Taipei 10002, Taiwan

Phone: +886-972651427

E-mail: [ywtien5106@ntu.edu.tw](mailto:ywtien5106@ntu.edu.tw)

**Supplementary Figure S1. The survival curves of PDAC patients classified by tumor grade and N stage in the TCR database.** The x-axis denotes the survival duration in months and the y-axis denotes the survival probability in percentage.


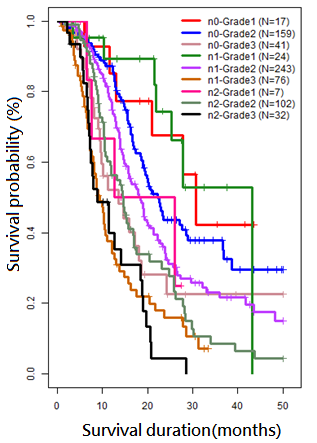


**Supplementary Figure S2. Survival outcomes based on the CART algorithm using tumor grade and N stage as input.** Four subgroups were generated from the two variables and their names, indicating the survival classification, are shown in red.


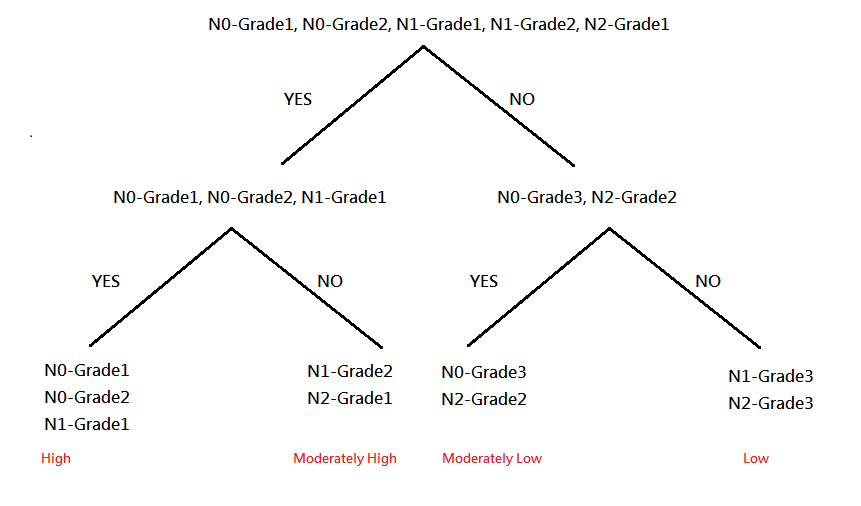


**Supplementary Figure S3. Average time-dependent AUC values calculated for years 1 – 4, for the proposed prognostic model using TCR data.** A 10-fold cross-validation (10CV) is conducted and each bar shows the average of the time dependent AUC over 10CV models.


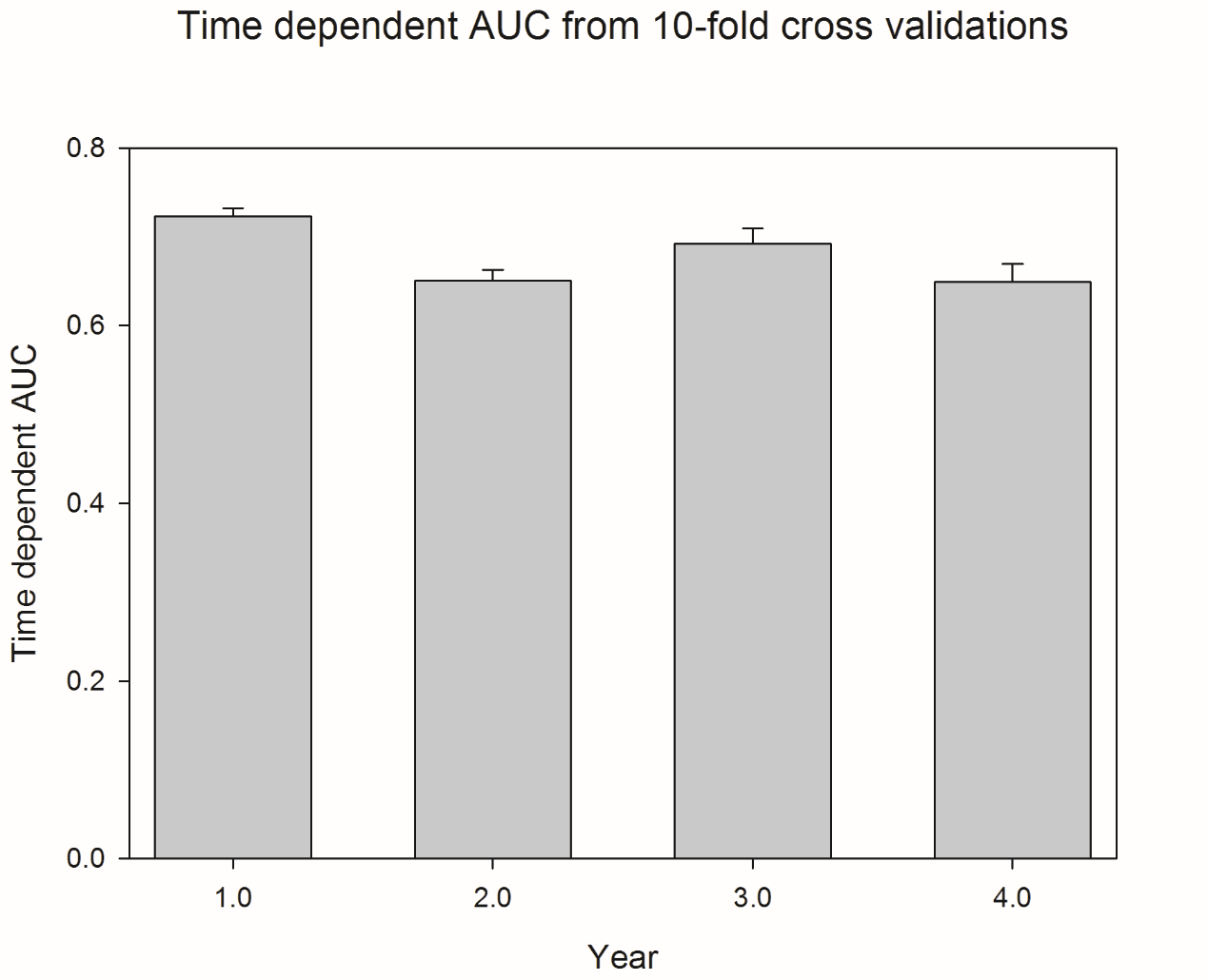


**Supplementary Figure S4. The survival curves of PDAC patients classified by tumor grade and N stage in the SEER database.** The x-axis denotes the survival duration in months and the y-axis denotes the survival probability in percentage.

**
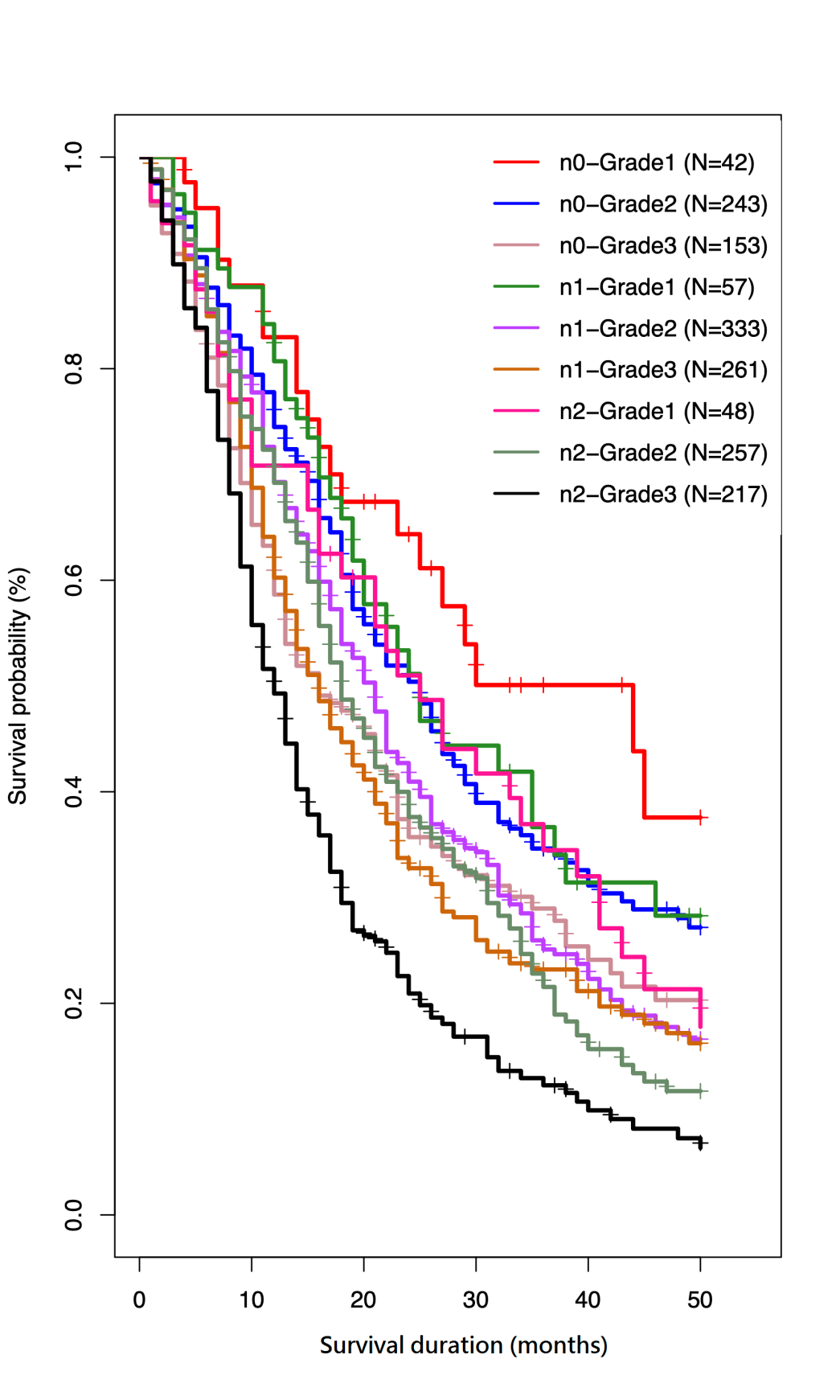
**

**Supplementary Figure S5.** **The survival curves of the 4 subgroups classified by N stage and grade in both the TCR (shown in blue) and the SEER (shown in red) datasets.** (A) subgroup High”, (B) subgroup “Moderately High”, (C) subgroup “Moderately Low”, (D) subgroup “Low”. The x-axis denotes the survival duration in months and the y-axis denotes the survival probability in percentage.
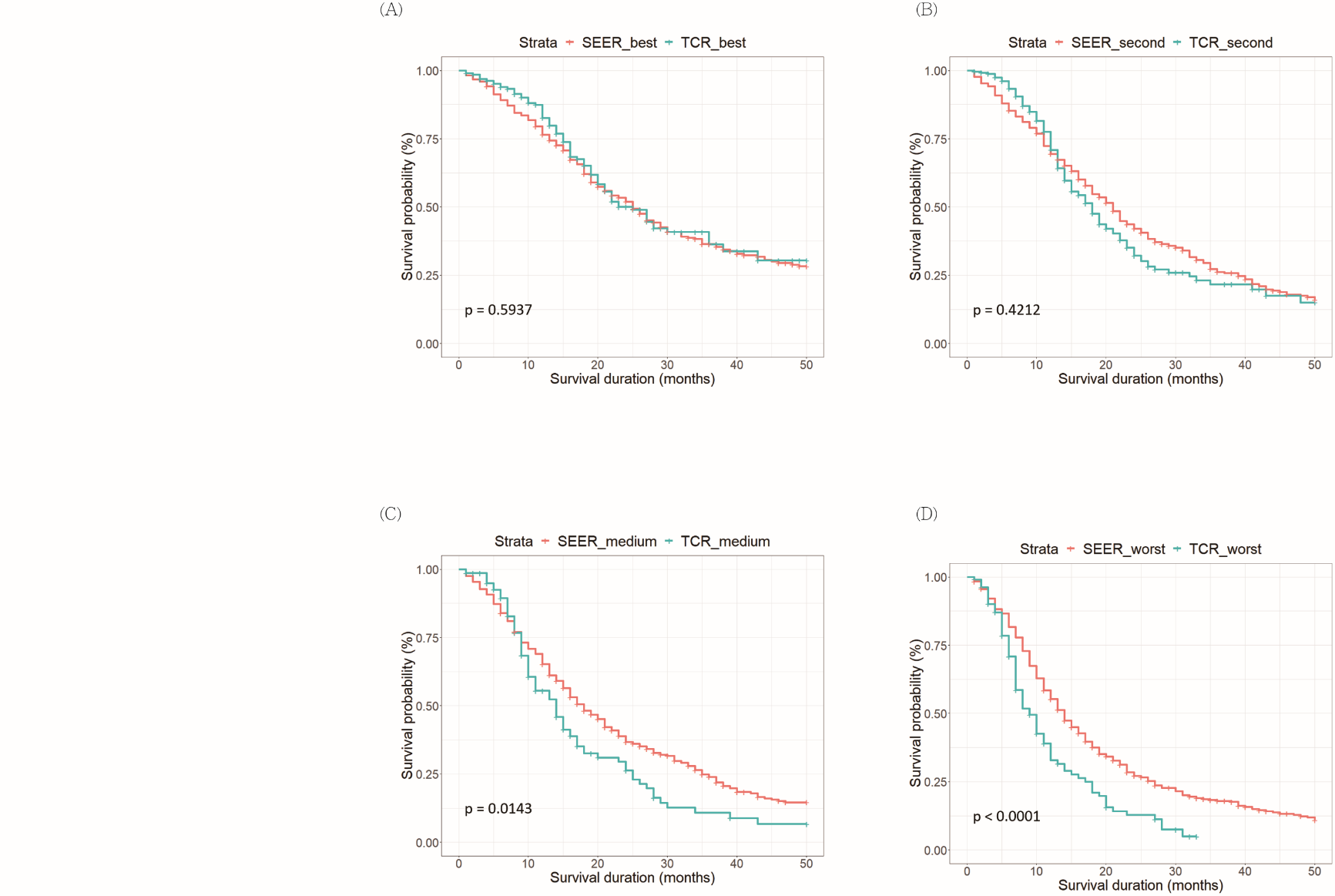


**Supplementary Table S1. Different definitions of the TNM model in PDAC patients in the AJCC7 and the AJCC8.**

|  | AJCC8 | AJCC7 |
| --- | --- | --- |
| T1 | Size ≤ 20 mm | Size ≤ 20 mm |
| T2 | 20 mm < size ≤ 40 mm | size > 20 mm |
| T3 | size > 40 mm | Tumor extends beyond the pancreas but without involvement of the celiac axis or the superior mesenteric artery |
| T4 | Tumor involves the celiac axis, common hepatic artery, or the superior mesenteric artery | Tumor involves the celiac axis or the superior mesenteric artery |
| N0 | No regional lymph node | No regional lymph node |
| N1 | Metastasis in 1-3 lymph nodes | Regional lymph node metastasis |
| N2 | Metastasis in ≥ 4 lymph nodes |  |
| M0 | No distant metastasis | |
| M1 | Distant metastasis | |

**Supplementary Table S2. The staging system of PDAC in AJCC8.**

| Stage | T | N | M |
| --- | --- | --- | --- |
| IA | T1 | N0 | M0 |
| IB | T2 | N0 | M0 |
| IIA | T3 | N0 | M0 |
| IIB | T1-3 | N1 | M0 |
| III | T_any_ | N2 | M0 |
|  | T4 | N_any_ | M0 |
| IV | T_any_ | N_any_ | M1 |

**Supplementary Table S3: The summary characteristics of the significant variables for the 701 patients from the Taiwan Cancer Registry.**

| Variable | Sample size (proportion) |
| --- | --- |
| Grade , N (%) |  |
| 1 | 48 (6.85%) |
| 2 | 504 (71.90%) |
| 3 | 143 (20.40%) |
| 4 | 6 (0.85%) |
| Lymph node involvement, N (%) |  |
| N0 | 217 (30.96%) |
| N1 | 343 (48.93%) |
| N2 | 141 (20.11%) |
| Adjuvant chemotherapy |  |
| With | 446 (63.62%) |
| Without | 255 (36.38%) |

**Supplementary Table S4. The interaction of tumor grade and N stage in the 701 stage III PDAC patients in the TCR database.**

|  | Grade 1 | Grade 2 | Grade 3 |
| --- | --- | --- | --- |
| N0 | 17 | 159 | 41 |
| N1 | 24 | 243 | 76 |
| N2 | 7 | 102 | 32 |

**Supplementary Table S5. The p-value of the log-rank test in the subgroups stratified by tumor grade and N stage in the TCR database.**

| Grade | N stage |  |  |
| --- | --- | --- | --- |
| Grade 1 |  | N0 | N1 |
|  | N1 | NS | - |
|  | N2 | NS | NS |
| Grade 2 |  | N0 | N1 |
|  | N1 | 0.013 | - |
|  | N2 | <0.0001 | 0.011 |
| Grade 3 |  | N0 | N1 |
|  | N1 | NS | - |
|  | N2 | NS | NS |

NS: non-significant.

**Supplementary Table S6. The statistics of the median survival duration and Cox hazard regression model of the 4 subgroups stratified by tumor grade and N stage in the SEER database.**

| Subgroup | N | Median survival duration (95% CI) | HR (95% CI) | P-value* |
| --- | --- | --- | --- | --- |
| High | 342 | 25 (22 - 29) | Ref | Ref |
| Moderately high | 381 | 21 (18 – 23) | 1.44 (1.20-1.72) | <0.0001 |
| Moderately low | 410 | 18 (16-21) | 1.56 (1.30-1.86) | <0.0001 |
| Low | 478 | 14 (13-15) | 1.98 (1.67-2.35) | <0.0001 |

* P-value was obtained from the Cox hazard regression model.

HR, hazard ratio; CI, confidence interval

The dependent variable in the regression model was death
